# Supplementary material for: Meal Timing Regulates the Human Circadian System
Source: Curr Biol. 2017 Jun 19;27(12):1768–1775.e3. doi: 10.1016/j.cub.2017.04.059 (PMC5483233; doi:10.1016/j.cub.2017.04.059)
Supplement: Document S1. Figures S1–S3 [file mmc1.pdf]

Current Biology, Volume *27*

Supplemental Information

Meal Timing Regulates the Human Circadian System

Sophie M.T. Wehrens, Skevoulla Christou, Cheryl Isherwood, Benita Middleton, Michelle A. Gibbs, Simon N. Archer, Debra J. Skene, and Jonathan D. Johnston

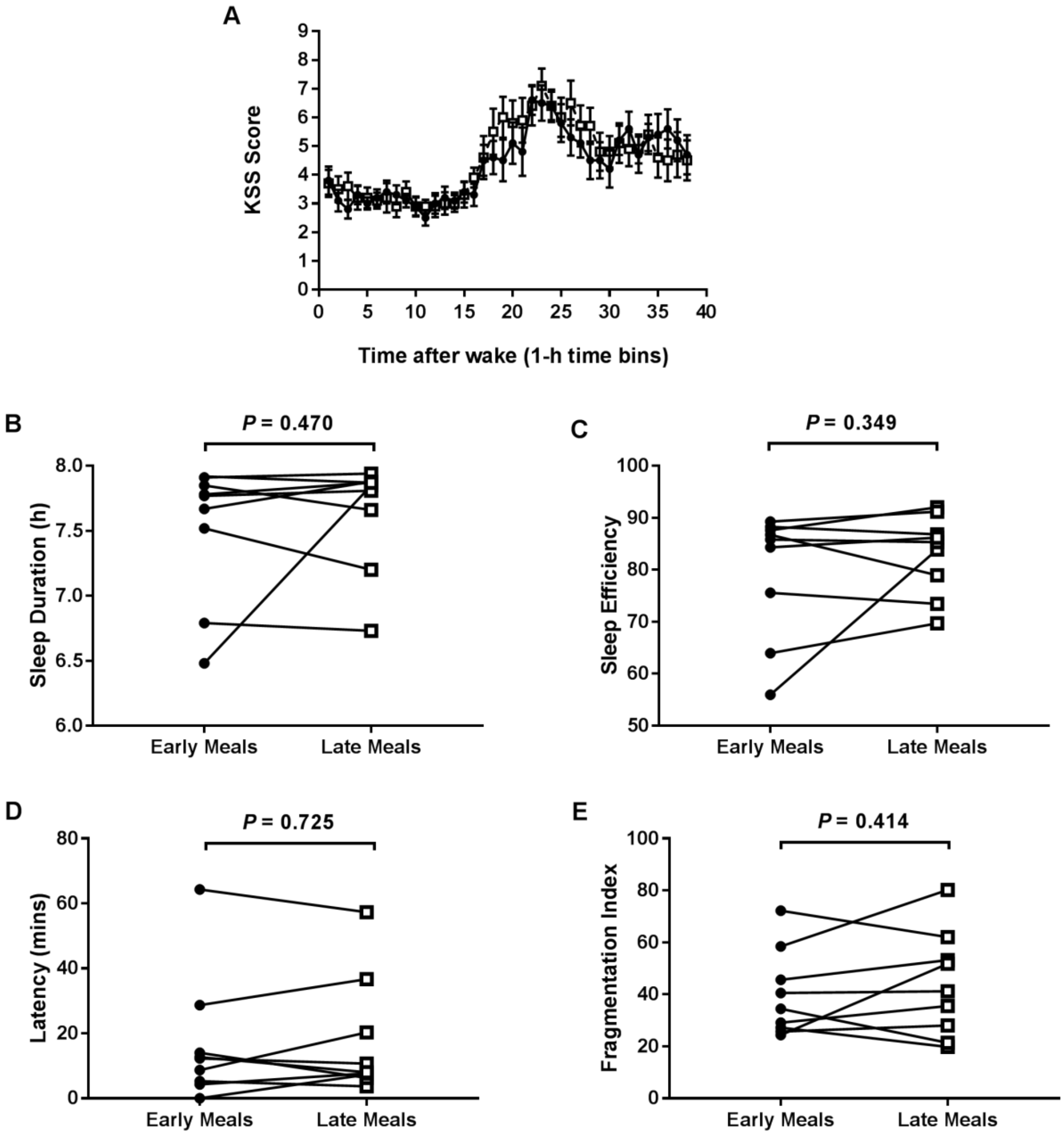

Figure S1. Sleep markers are not altered by a 5-hour delay in meal times (related to Figure 1).
(A) Subjective sleepiness was determined by hourly completion of the Karolinska Sleepiness Scale (KSS) during constant routine conditions. Black circles with solid lines represent data following early meals (0.5, 5.5 and 10.5 hours after waking up). White squares with dashed lines represent data following a 5-hour delay in each meal. There was a significant effect of time ($F_{(37,333)} = 10.84$, $p < 0.001$) , but no effect of meals ($F_{(1,9)} = 0.36$, $p = 0.562$) or meal x time interaction ($F_{(37,333)} = 1.14$, $p = 0.274$; 2-way repeated measures ANOVA). Data are mean ± SEM, n = 10, plotted as KSS score relative to each individual's time since wake.
(B-E) Actigraphy analysis of sleep periods following early and late meals. Data are from n = 9 participants and analysed by paired t-test. There was no significant effect on (B) sleep duration ($t_{(8)} = 0.759$, $p = 0.470$). (C) sleep efficiency ($t_{(8)} = 0.995$, $p = 0.349$). (D) sleep latency ($t_{(8)} = 0.365$, $p = 0.725$). or (E) fragmentation index ($t_{(8)} = 0.862$, $p = 0.414$).

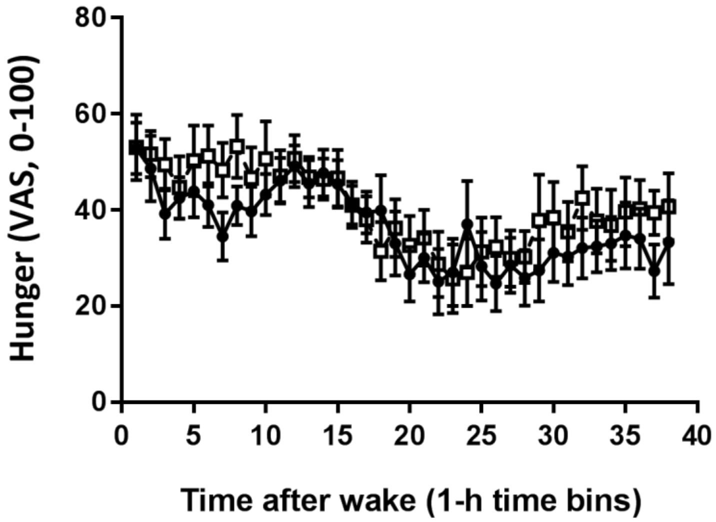

Figure S2. Subjective hunger is not altered by a 5-hour delay in meal times (related to Figure 1). Hunger was determined by hourly completion of a hunger visual analogue scale (VAS) during constant routine conditions. Black circles with solid lines represent data following early meals (0.5, 5.5 and 10.5 hours after waking up). White squares with dashed lines represent data following a 5-hour delay in each meal. There was a significant effect of time ($F_{(37,333)} = 6.12$, $p < 0.001$), but no significant effect of meal ($F_{(1,9)} = 1.60$, $p = 0.238$) or meal x time interaction ($F_{(37,333)} = 1.03$, $p = 0.424$; 2-way repeated measures ANOVA). Data are mean ± SEM, n = 10 , plotted as hunger score relative to each individual's time since wake.

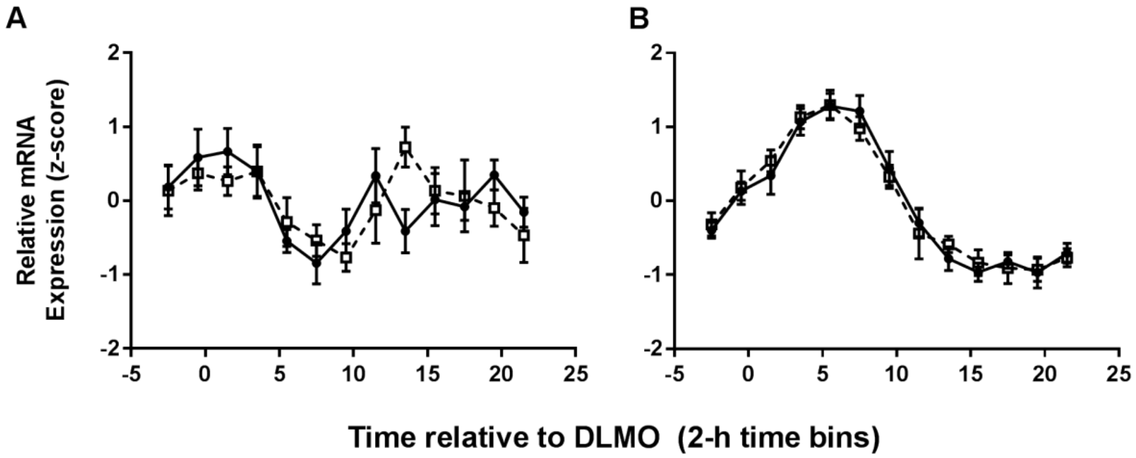

Figure S3. Clock gene rhythms in whole blood are not altered by a 5-hour delay in meal times (related to Figure 3).
Temporal expression profiles of (A) *BMAL1* and (B) *PER3* in 2-hourly blood leukocyte samples collected in constant routine conditions. Black circles with solid lines represent data following early meals (0.5, 5.5 and 10.5 hours after waking up). White squares with dashed lines represent data following a 5-hour delay in each meal. Data are mean ± SEM, n = 10, plotted as z-scored relative mRNA expression relative to each individual's dim light melatonin onset (DLMO).
