# Supplementary material for: Meal Timing Regulates the Human Circadian System
Source: Curr Biol. 2017 Jun 19;27(12):1768–1775.e3. doi: 10.1016/j.cub.2017.04.059 (PMC5483233; doi:10.1016/j.cub.2017.04.059)
Supplement: Document S2. Article plus Supplemental Information [file mmc2.pdf]

Report

Current Biology

Meal Timing Regulates the Human Circadian System

Highlights

- A 5-hr delay in meal times changes the phase relationship of human circadian rhythms
- Plasma glucose, but not insulin or triglyceride, rhythms are delayed by late meals
- Adipose *PER2* rhythms are delayed by late meals
- Rhythm changes occur without altered subjective or actigraphic sleep markers

Authors

Sophie M.T. Wehrens,
Skevoulla Christou,
Cheryl Isherwood, ..., Simon N. Archer,
Debra J. Skene, Jonathan D. Johnston

Correspondence

j.johnston@surrey.ac.uk

In Brief

Wehrens et al. measure the effect of a 5-hr meal delay on the human circadian system. Late meals delay rhythms of plasma glucose and adipose *PER2* clock gene expression. Meal timing may help to reset the circadian system in cases of shift work, jet lag, and circadian rhythm disorders.

CrossMark

Wehrens et al., 2017, Current Biology *27*, 1768–1775
June 19, 2017 © 2017 The Authors. Published by Elsevier Ltd.
http://dx.doi.org/10.1016/j.cub.2017.04.059

CellPress

Current Biology
Report

Meal Timing Regulates the Human Circadian System

Sophie M.T. Wehrens,[1] Skevoulla Christou,[1] Cheryl Isherwood,[1] Benita Middleton,[1] Michelle A. Gibbs,[1] Simon N. Archer,[1] Debra J. Skene,[1] and Jonathan D. Johnston[1,2,*]
[1]Faculty of Health and Medical Science, University of Surrey, Stag Hill Campus, Guildford, Surrey GU2 7XH, UK
[2]Lead Contact
*Correspondence: j.johnston@surrey.ac.uk
http://dx.doi.org/10.1016/j.cub.2017.04.059

SUMMARY

Circadian rhythms, metabolism, and nutrition are intimately linked [1, 2], although effects of meal timing on the human circadian system are poorly understood. We investigated the effect of a 5-hr delay in meals on markers of the human master clock and multiple peripheral circadian rhythms. Ten healthy young men undertook a 13-day laboratory protocol. Three meals (breakfast, lunch, dinner) were given at 5-hr intervals, beginning either 0.5 (early) or 5.5 (late) hr after wake. Participants were acclimated to early meals and then switched to late meals for 6 days. After each meal schedule, participants' circadian rhythms were measured in a 37-hr constant routine that removes sleep and environmental rhythms while replacing meals with hourly isocaloric snacks. Meal timing did not alter actigraphic sleep parameters before circadian rhythm measurement. In constant routines, meal timing did not affect rhythms of subjective hunger and sleepiness, master clock markers (plasma melatonin and cortisol), plasma triglycerides, or clock gene expression in whole blood. Following late meals, however, plasma glucose rhythms were delayed by 5.69 ± 1.29 hr ($p < 0.001$), and average glucose concentration decreased by 0.27 ± 0.05 mM ($p < 0.001$). In adipose tissue, *PER2* mRNA rhythms were delayed by 0.97 ± 0.29 hr ($p < 0.01$), indicating that human molecular clocks may be regulated by feeding time and could underpin plasma glucose changes. Timed meals therefore play a role in synchronizing peripheral circadian rhythms in humans and may have particular relevance for patients with circadian rhythm disorders, shift workers, and transmeridian travelers.

RESULTS

No Change in Rhythms of SCN Clock-Driven Hormones, Markers of Sleep, or Subjective Appetite

Mammalian circadian rhythms are driven by a master clock, within the suprachiasmatic nuclei (SCN) of the hypothalamus, and peripheral clocks located throughout the body [3]. For the circadian system to function optimally, individual clocks must be correctly synchronized to one another and to the external environment. Abnormal circadian rhythms or defects in synchronization pathways can result in circadian misalignment or desynchrony, which are associated with poor health and metabolic disorders [4, 5]. In most individuals, the SCN clock is set to solar time by photic input pathways originating in the retina [6]; the SCN then synchronize peripheral clocks through neuronal pathways, hormone rhythms, core body temperature, and behaviors such as the cycle of feeding and fasting [3]. Photic cues are of primary importance for resetting human rhythms [7]. Regularly timed non-photic cues, however, can regulate rhythms in non-human species; for example, temporal restriction of food availability resets the phase of rodent peripheral clocks [8, 9], with more subtle effects on the rodent SCN [10]. Human studies have revealed that post-prandial responses are dependent on meal timing [11–14], but little is known of the ability of meals per se to alter the timing of human circadian rhythms.

We investigated a 5-hr delay in three isocaloric daily meals (breakfast, lunch, and dinner) with identical macronutrient content on circadian rhythms using a 13-day laboratory protocol (Figure 1A). The overarching hypothesis was that the delay in meal timing delays peripheral rhythms, but not markers of the SCN clock. Phase changes were indirectly assessed by meal × time-of-day interactions in ANOVA analysis of grouped data and directly assessed by cosinor analysis of individual participant data (see STAR Methods).

We first measured the effect of meal time on plasma melatonin and cortisol rhythms, which are well-validated markers of the SCN clock. No significant changes were found in the temporal profiles of either hormone (Figures 1B and 1C). Next, in each individual, melatonin phase was measured using the dim light melatonin onset (DLMO), and cortisol acrophase was calculated using cosinor analysis. Delayed meals had no significant effect on either DLMO or cortisol phase (DLMO: $t_{(9)} = 0.94$, $p = 0.372$; cortisol: $t_{(9)} = 0.96$, $p = 0.182$; paired t test).

As sleep disruption is known to modulate metabolic physiology [15], we assessed subjective sleepiness throughout each constant routine using the Karolinska Sleepiness Scale (KSS). The expected temporal variation was observed, but there was no significant effect of meal timing (Figure S1). Furthermore, we were unable to detect any effects of meal timing on objective markers of sleep assessed by actigraphy (Figure S1). We assessed the influence of meal time on subjective appetite using a visual analog scale (VAS) but again found no significant effect (Figure S2).

This is an open access article under the CC BY license (http://creativecommons.org/licenses/by/4.0/).

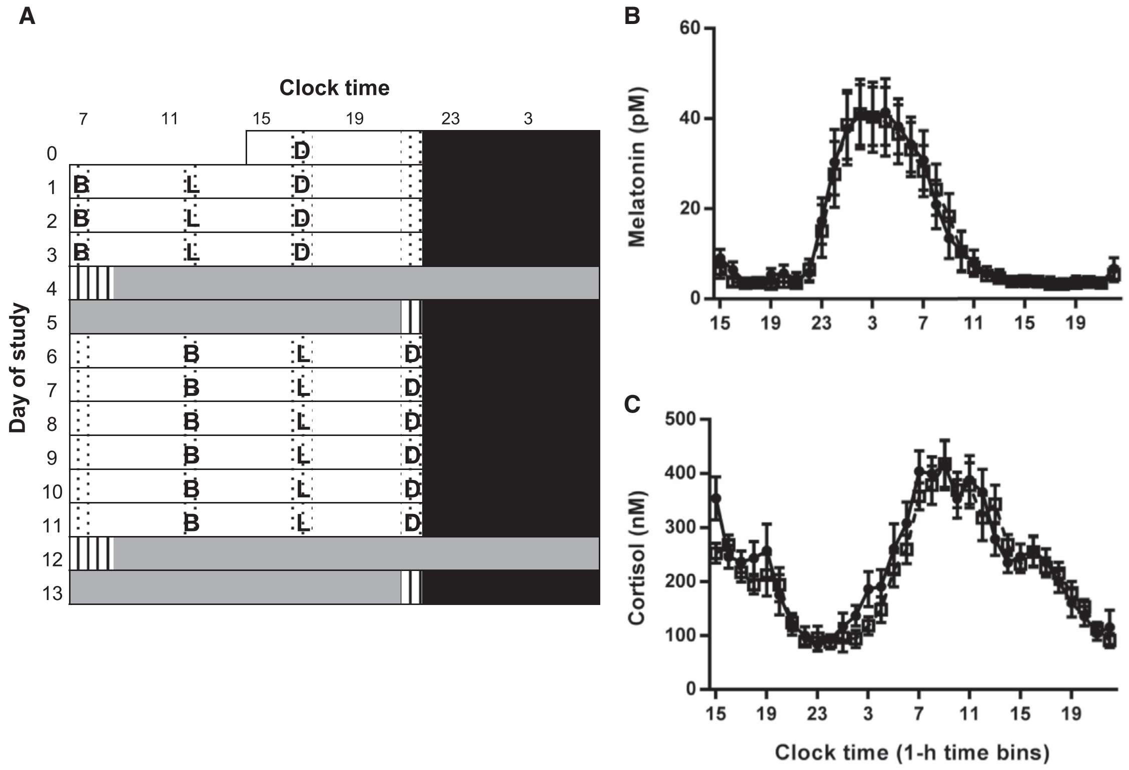

Figure 1. Study Protocol and Phase of SCN-Driven Hormone Rhythms

(A) In order to maximize circadian entrainment prior to beginning the study protocol, participants maintained a self-selected pre-laboratory light-dark and sleep-wake pattern based on their habitual routine for 10 days. During the last week of the pre-laboratory period they ate breakfast (B) 30 min after wake, lunch (L) 5.5 hr after wake, and dinner (D) 10.5 hr after wake. Participants then entered the laboratory on day 0. During days 0–3, participants remained on their self-selected sleep-wake cycle. They slept in individual bedrooms in darkness (0 lux; black bars) and were awake in bright room light (~500 lux in direction of gaze) during the day. Waking time was spent in communal areas (white bars) and in individual rooms (dotted bars). Isocaloric meals (B, L, D) were given 0.5, 5.5, and 10.5 hr after waking up, matching the week of pre-laboratory meal timing. On day 4, participants began a 37-hr constant routine in individual rooms (<8 lux; gray bars). Participants had a standard night's sleep on day 5, before 6 more days of the sleep-wake and light-dark cycles (days 6–11). Conditions were equal to days 1–3 except for a 5-hr delay in all meal times. A second constant routine then commenced on day 12. Immediately before and after each constant routine, participants were kept in a constant routine-like environment but allowed to move within their rooms (hatched bars).

(B and C) Concentration of melatonin (B) and cortisol (C) in hourly plasma samples collected in constant routine conditions. Black circles with solid lines represent data following early meals (0.5, 5.5, and 10.5 hr after waking up). White squares with dashed lines represent data following a 5-hr delay in each meal. Two-way repeated-measures ANOVA revealed a significant effect of time (melatonin: $F_{(31, 279)} = 19.00$, $p < 0.001$; cortisol: $F_{(31, 279)} = 20.31$, $p < 0.001$), but no significant effect of meals (melatonin: $F_{(1, 9)} = 2.97$, $p = 0.119$; cortisol: $F_{(1, 9)} = 2.27$, $p = 0.166$) or meal × time interaction (melatonin: $F_{(31, 279)} = 0.13$, $p = 0.124$; cortisol: $F_{(31, 279)} = 1.39$, $p = 0.090$). Data are mean ± SEM, n = 10. Statistical significance is defined as $p < 0.01$ (following Bonferroni correction for analysis of a total of five rhythmic plasma markers).

See also Figures S1 and S2.

Plasma Glucose, but Not Insulin or Triglyceride, Rhythms Are Affected by Meal Time

Plasma glucose concentration exhibited significant effects of time of day, meals, and meal × time-of-day interaction (Figure 2A). In order to quantify the effect of timed meals on glucose rhythm phase, we used cosinor analysis. The glucose acrophase occurred 1.31 ± 0.82 hr before DLMO following early meals but 4.38 ± 0.83 hr after DLMO following late meals. The 5-hr change in meal time delayed the relative phase of glucose rhythms by 5.69 ± 1.29 hr (Figure 2D).

The possible contribution of insulin to the delayed glucose rhythms was also investigated. Despite a significant effect of time of day, there was no significant effect of meals or meal × time-of-day interaction on plasma insulin concentration (Figure 2B). Cosinor analysis estimated an insulin acrophase 7.99 ± 1.99 hr after DLMO following early meals and 11.36 ± 0.89 hr after DLMO following late meals (Figure 2E).

There was a significant effect of time of day, but no significant effect of meals or meal × time-of-day interaction on plasma triglyceride concentration (Figure 2C). Cosinor analysis estimated a triglyceride acrophase 6.59 ± 0.62 hr after DLMO following early meals and 7.38 ± 0.30 hr after DLMO following late meals (Figure 2F).

Differential Response of Clock Gene Rhythms in White Adipose Tissue and Blood

To test the hypothesis that delayed meals delay molecular circadian rhythms in peripheral tissues, we measured clock gene transcripts in serial biopsies of white adipose tissue (WAT) using a refinement of our previously published protocol [16, 17]. Gene

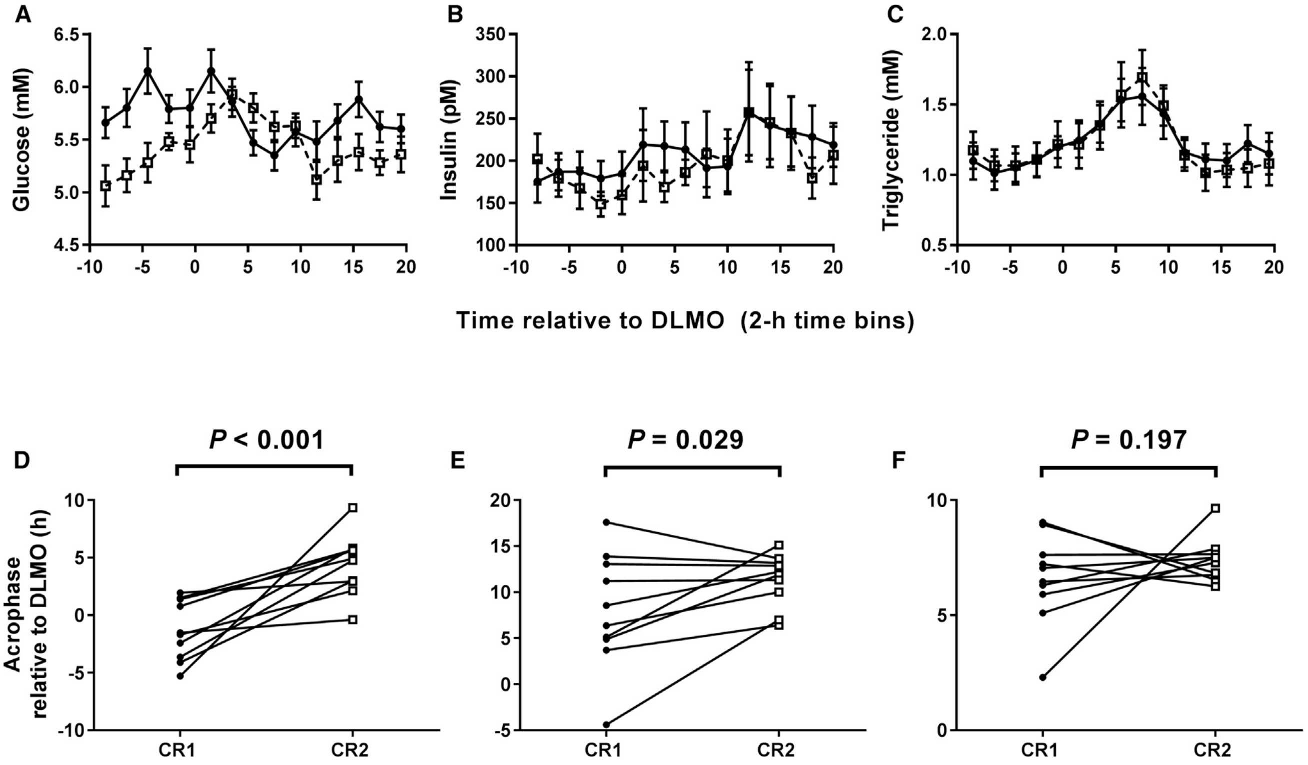

Figure 2. A 5-hr Delay in Meal Times Delays the Plasma Glucose Circadian Rhythm

(A–C) Concentration of glucose (A), insulin (B), and triglyceride (C) in 2-hourly plasma samples collected in constant routine conditions. Data are plotted as mean ± SEM. Black circles with solid lines represent data following early meals (0.5, 5.5, and 10.5 hr after waking up). White squares with dashed lines represent data following a 5-hr delay in each meal.

(A) There were significant effects of time ($F_{(14,126)} = 3.71$, $p < 0.001$), meals ($F_{(1, 9)} = 29.84$, $p < 0.001$), and meal × time interaction ($F_{(14,126)} = 5.10$, $p < 0.001$) on glucose concentration.

(B) There was a significant effect of time ($F_{(14,126)} = 2.79$, $p = 0.001$), but no significant effect of meals ($F_{(1, 9)} = 4.69$, $p = 0.059$) or meal × time interaction ($F_{(14,126)} = 1.16$, $p = 0.312$) on plasma insulin concentration.

(C) There was a significant effect of time ($F_{(14,126)} = 18.44$, $p < 0.001$), but no significant effect of meals ($F_{(1, 9)} = 0.01$, $p = 0.913$) or meal × time interaction ($F_{(14,126)} = 1.19$, $p = 0.294$) on plasma triglyceride concentration.

(D–F) Acrophase of glucose (D), insulin (E), and triglyceride (F) rhythms in individuals following early meals (constant routine 1, CR1; black circles) and following a 5-hr delay in meal time (constant routine 2, CR2; white squares). Using a paired t test, there was a significant effect of meal timing on glucose phase (delay of 5.59 ± 1.29 hr; $t_{(9)} = 4.415$, $p < 0.001$), but not on the phase of insulin ($t_{(9)} = 2.179$, $p = 0.029$; note Bonferroni-corrected critical p value below) or triglyceride ($t_{(9)} = 0.896$, $p = 0.197$).

(A–F) Data are from n = 10 participants, calculated relative to each individual's dim light melatonin onset (DLMO). Statistical significance is defined as $p < 0.01$ (following Bonferroni correction for analysis of a total of five rhythmic plasma markers).

expression was measured in the seven participants from whom we were able to obtain five biopsies, one every 6 hr, in both constant routines. Data were obtained by RT-PCR for three canonical clock genes and *Z* scored prior to analysis (Figures 3A–3C). There was a significant effect of time of day, but no overall effect of meals on all three genes. There was a significant meal × time-of-day interaction for *PER2*, but not for *PER3* or *BMAL1*. Cosinor analysis also revealed a significant effect of meal timing on *PER2* phase, but not on the phase of *PER3* or *BMAL1* (Figures 3D–3F).

We next studied clock gene rhythmicity in whole blood samples. Consistent with previously published constant routine data [18], we found weak rhythms in *BMAL1* (Figure S3A) and robust rhythms in *PER3* gene expression (Figure S3B). However, no significant effect of delayed meals on either rhythm was observed.

Reduced Glucose Concentration Following Late Meals

Two-way repeated-measures ANOVA analysis of the time series data indicated a significant decrease in glucose concentration in the constant routine following late meals (Figure 2A). To investigate this in more detail, we compared each participant's mean glucose concentration in the two constant routines. There was a significant decrease in the mean glucose concentration following late meals (Figure 4A), with all ten of the participants exhibiting lower plasma glucose after late meals. There was, however, no significant decrease in the mean concentration of plasma insulin (Figure 4B) or triglyceride (Figure 4C) following late meals. We next compared the peak and trough values for each participant in each constant routine to determine whether the lower glucose concentration was due to a reduced peak, trough, or both (Figure 4D). There was an overall effect of meals and a significant difference between peak and trough values. There was

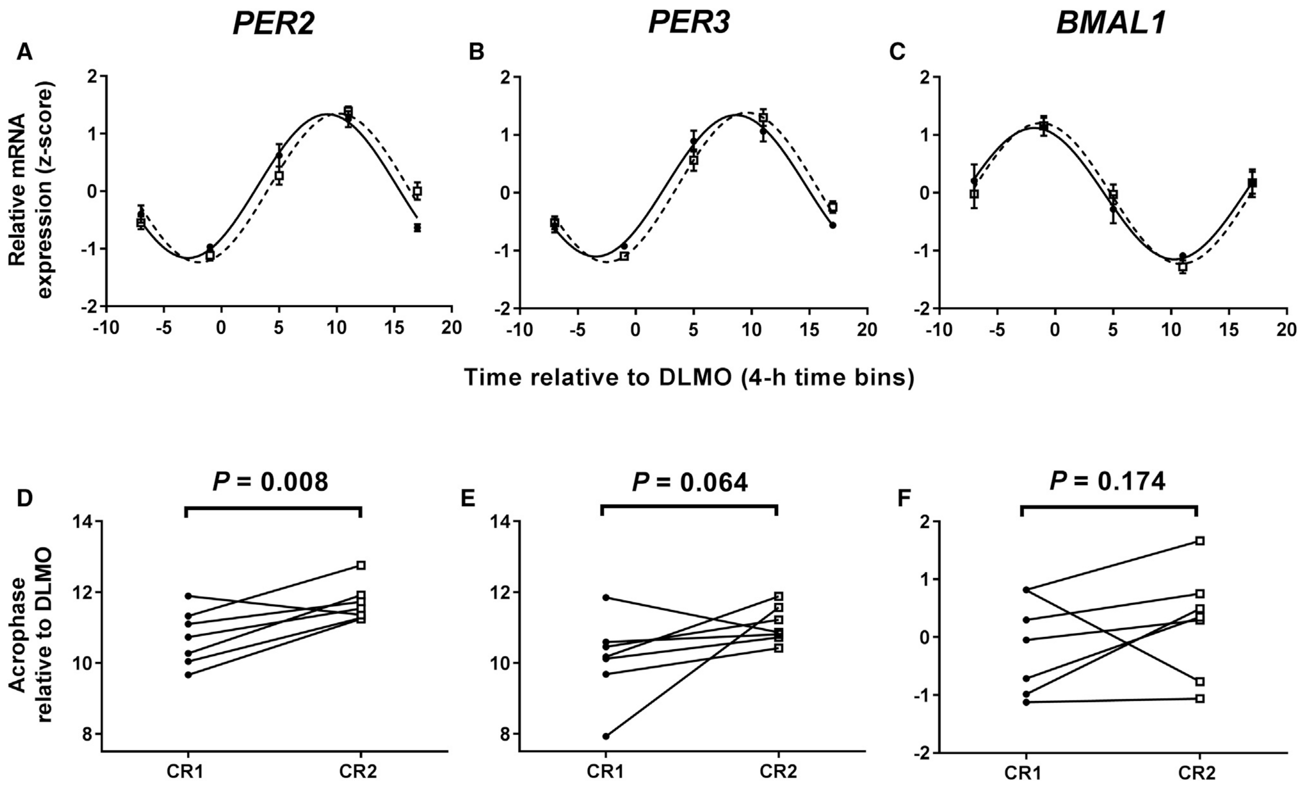

Figure 3. A 5-hr Delay in Meal Times Delays Clock Gene Rhythms in White Adipose Tissue

(A–C) Temporal expression profiles of *PER2* (A), *PER3* (B), and *BMAL1*(C) in 6-hourly white adipose tissue biopsies collected in constant routine conditions. Data are plotted as mean ± SEM. Black circles with solid lines represent data following early meals (0.5, 5.5, and 10.5 hr after waking up). White squares with dashed lines represent data following a 5-hr delay in each meal. Two-way repeated-measures ANOVA revealed a significant effect of time for *PER2* ($F_{(4,24)} = 56.81$, $p < 0.001$), *PER3* ($F_{(4,24)} = 65.67$, $p < 0.001$), and *BMAL1* ($F_{(4,24)} = 21.44$, $p < 0.001$). There was no overall effect of meal for any gene: *PER2* ($F_{(1,6)} = 1.00$, $p = 0.356$), *PER3* ($F_{(1,6)} = 1.07$, $p = 0.340$), and *BMAL1* ($F_{(1,6)} = 1.08$, $p = 0.339$). There was a significant meal × time interaction for *PER2* ($F_{(4,24)} = 7.31$, $p < 0.001$), but not for *PER3* ($F_{(4,24)} = 2.44$, $p = 0.075$) or *BMAL1* ($F_{(4,24)} = 0.58$, $p = 0.680$).

(D–F) Acrophase of *PER2* (D), *PER3* (E), and *BMAL1* (F) rhythms in individuals following early meals (CR1; black circles) and following a 5-hr delay in meal time (CR2; white squares). Using a paired t test, there was a significant effect of meal timing on *PER2* phase (delay of 0.97 ± 0.29 hr; $t_{(6)} = 3.35$, $p = 0.008$), but not on the phase of *PER3* ($t_{(6)} = 1.77$, $p = 0.064$) or *BMAL1* ($t_{(6)} = 1.02$, $p = 0.174$).

(A–F) Data are from n = 7 participants, calculated relative to each individual's DLMO. Statistical significance is defined as $p < 0.017$ (following Bonferroni correction for analysis of a total of three rhythmic adipose markers).

See also Figure S3.

no significant interaction between the two factors, however, indicating a similar lowering of both peak and trough plasma glucose following the late meals.

DISCUSSION

This report demonstrates that meal timing exerts a variable influence over human physiological rhythms, with notable changes occurring in aspects of glucose homeostasis. A 5-hr delay in meal times induced a comparable delay in the phase of circadian plasma glucose rhythms, as assessed under constant routine conditions. These altered glucose rhythms were accompanied by a 1-hr delay in the phase of WAT *PER2* rhythms, but no change in markers of the SCN clock (melatonin, cortisol), rhythms of plasma insulin and triglyceride, or clock gene rhythms in whole blood. We also observed a reduction in plasma glucose concentration during the constant routine following late meals.

To limit our intervention to meal timing, participants maintained identical light-dark and sleep-wake schedules on days when timed meals were given. Sample collection then occurred in constant routine conditions after both early and late meals. Constant routines remove environmental fluctuations and sleep and replace meals with equally spaced isocaloric snacks [19]; the rhythms obtained are thus the product of endogenous circadian processes and not the result of acute post-prandial responses. Subjective sleepiness and hunger exhibited the expected temporal patterns. Sleepiness increased over the course of each constant routine, due to continuous wakefulness, and was highest during the subjective night. Self-reported hunger scores dipped in the early subjective morning, as observed by others [20, 21]. Meal timing had no effect on these subjective sleep and appetite markers, or actigraphic sleep parameters recorded prior to each constant routine, indicating that responses to a shift in meal times are unlikely to be driven by changes in sleep propensity or appetite.

Circadian regulation of plasma glucose and triglyceride concentration in humans has been reported by others using constant routine [12, 13, 22] and forced desynchrony [14] protocols.

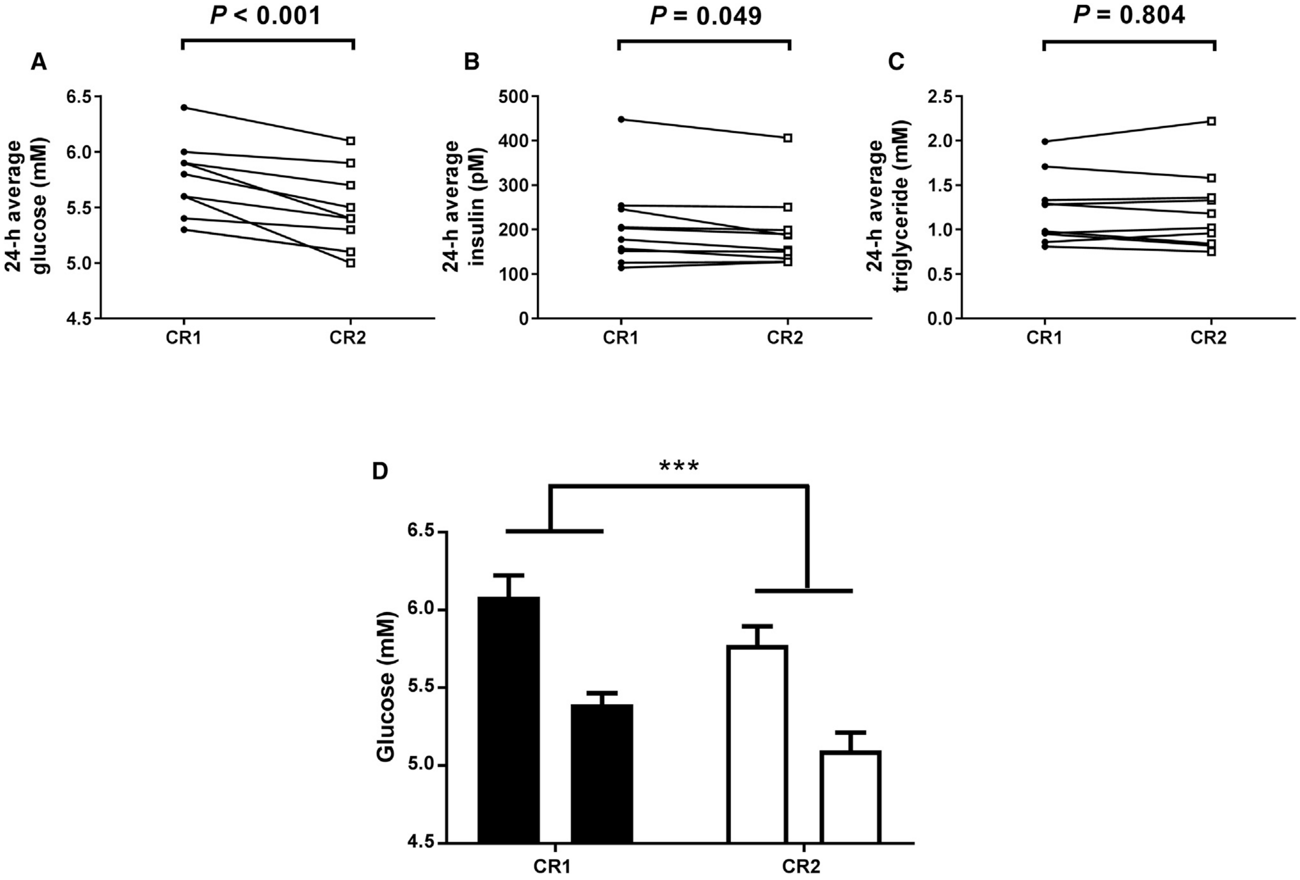

Figure 4. The Average Plasma Glucose Concentration in Constant Routine Conditions Is Reduced Following a 5-hr Delay in Meal Times

(A–C) 24-hr average concentration of glucose (A), insulin (B), and triglyceride (C) in plasma samples collected in constant routine conditions following early meals (CR1; black circles) and following a 5-hr delay in meal time (CR2; white squares). There was a significant decrease in the mean glucose concentration following late meals (5.45 ± 0.11 mmol/L) compared to early meals (5.72 ± 0.11 mmol/L, $t_{(9)} = 5.22$, $p < 0.001$, paired t test). Following Bonferroni correction of the critical p value, there was no significant decrease in the mean concentration of plasma insulin following late meals (208.2 ± 30.46 versus 192.6 ± 26.75 pmol/L, early versus late, respectively; $t_{(9)} = 2.27$, $p = 0.049$, paired t test). There was no significant difference in mean triglyceride concentration (1.22 ± 0.12 versus 1.21 ± 0.14 mmol/L, early versus late meals, respectively; $t_{(9)} = 0.26$, $p = 0.804$, paired t test).

(D) Peak and trough concentration of glucose in plasma samples collected in constant routine conditions following early meals (black bars) and a 5-hr delay in meal time (white bars). Using two-way repeated-measures ANOVA, there was an overall significant effect of meals ($F_{(1, 9)} = 22.98$, $p = 0.001$), a significant difference between peak and trough values ($F_{(1, 9)} = 177.6$, $p < 0.001$), but no significant interaction between the two factors ($F_{(1, 9)} = 0.01$, $p = 0.914$). ***$p < 0.001$ (early meals/CR1 versus late meals/CR2). Data are plotted as mean ± SEM.

(A–D) Statistical significance is defined as $p < 0.01$ (following Bonferroni correction for analysis of plasma concentration in five markers). Data are from n = 10 participants.

In addition, one study has reported minor (~1 hr) phase shifts of human temperature and heart rate rhythms following morning or evening carbohydrate-rich meals, but with no effect on melatonin timing [23]. Very little research has addressed how temporal aspects of feeding regulate the circadian system of humans, however. The altered glucose rhythms in our study did not coincide with changes in plasma insulin rhythms and could thus be driven by altered rhythms of insulin sensitivity and/or glucose release from storage tissues. We observed no change in plasma triglyceride rhythm. Meal timing therefore appears to exert greater control over glucose homeostasis than lipid metabolism and can dissociate the temporal regulation of these key physiological processes.

We also investigated the effect of meal timing on markers of both central and peripheral circadian clocks. On the basis of previous animal and human experiments, we hypothesized that meal time would not alter the phase of melatonin and cortisol rhythms, reliable markers of the SCN clock. Clock gene rhythms in the SCN of rodents permitted ad libitum quantities of food do not synchronize to meal time [8, 9]. Furthermore, melatonin and cortisol rhythms in totally blind humans do not readily entrain to ad libitum non-photic cues [24]. Our melatonin and cortisol data demonstrated no differences after early compared to late meals. This suggests that the observed changes in rhythms of metabolic parameters are SCN independent, presumably occurring via effects on peripheral clocks.

Data from animal studies indicate that circadian clocks in multiple peripheral tissues contribute to glucose homeostasis [25–30]. We therefore tested the hypothesis that late meals delay the phase of human peripheral clocks. We and others have

previously demonstrated robust gene expression rhythms in serial WAT biopsies and blood samples [16, 18, 31, 32]. Here we observed a significant 1-hr delay in WAT *PER2* expression. Although this change is smaller than the phase delay of plasma glucose rhythms, it nonetheless indicates for the first time that feeding patterns may be capable of synchronizing human peripheral clocks. Based on the differential resynchronization rate of murine clocks to food [8, 9], we predict that the effect of meal time on clocks in other peripheral tissues involved in glucose homeostasis (e.g., liver, pancreas) would be larger than in WAT. Indeed, tissue-specific responsiveness of peripheral tissue clocks is demonstrated by the lack of shift in *PER3* rhythms in our blood samples.

Mean concentration of plasma glucose was 0.27 mM (4.7%) lower following late meals. The reduction of both peak and trough concentrations implies lower plasma glucose across the circadian cycle, with no change in rhythm amplitude. The cause of this change is unknown, but may involve the uncoupling of clocks in tissues that regulate glucose metabolism. Alternatively, experimental design may have resulted in an order effect on glucose, but not triglyceride, concentration. Order effects are extremely unlikely to contribute to the reported phase delays, however, as metabolite and gene expression data were analyzed relative to each individual's endogenous melatonin phase. It is currently unclear how plasma glucose concentrations in a constant routine, where participants receive small hourly snacks, relate to the elevated post-prandial glucose excursion that occurs in the biological evening and night, compared to the early morning [12, 13]. These questions will be the focus of future research.

Limitations of the current study include the restricted participant demographics (all young men) and the fact that it is impossible to serially biopsy most human tissues closely associated with glucose homeostasis. The use of tightly controlled demographics is standard for this type of human laboratory trial. However, now that we have identified physiological responses in young male volunteers, it will be possible to target future studies to other groups. Serial sampling of human tissues has obvious practical considerations, limiting the number of study participants and the sampling resolution. Use of our WAT biopsy protocol has nonetheless enabled us to uncover novel effects of meal timing on gene expression rhythms in a metabolically important human tissue.

Our study reveals clear effects of meal timing on glucose homeostasis in a controlled laboratory setting. It is possible that timed meals could have a different effect on individuals not as tightly entrained as our study participants. Nonetheless, the implications of this novel finding include insight into the effects of eating behavior on human physiology, e.g., in patients with night eating disorder. The most wide-ranging impact, however, could be an addition to the existing light and sleep strategies for treating people with circadian desynchrony, which occurs following shift work and transmeridian flight. Prolonged desynchrony and shift work have been associated with obesity and cardiometabolic disease, so measures to appropriately synchronize the circadian system could benefit long-term health in many people. Timed interventions such as light exposure, or administration of oral agents including melatonin and caffeine, regulate the phase of human SCN-driven hormonal rhythms [33–37]. Oral administration of glucocorticoid can also phase shift clock gene rhythms in human blood mononuclear cells [38]. We now provide a non-pharmacological means by which some peripheral metabolic rhythms can be phase shifted in humans. Future work will need to examine the effects of timed meal patterns in simulated and real-life models of human jet lag and shift work. Animal studies [39] indicate this could be a very fruitful area of research.

STAR★METHODS

Detailed methods are provided in the online version of this paper and include the following:

- KEY RESOURCES TABLE
- CONTACT FOR REAGENT AND RESOURCE SHARING
- EXPERIMENTAL MODEL AND SUBJECT DETAILS
- METHOD DETAILS
  - Pre-laboratory study period
  - Laboratory study design
  - Biopsy and blood sample collection
  - Actigraphy measurements
  - Plasma hormone and metabolite measurements
  - Gene expression measurements
- QUANTIFICATION AND STATISTICAL ANALYSIS

SUPPLEMENTAL INFORMATION

Supplemental Information includes three figures and can be found with this article online at http://dx.doi.org/10.1016/j.cub.2017.04.059.

AUTHOR CONTRIBUTIONS

S.M.T.W., M.A.G., S.N.A., D.J.S., and J.D.J. carried out study design. C.I. carried out diet design. S.M.T.W., S.C., and C.I. handled data collection. S.M.T.W., S.C., C.I., B.M., S.N.A., D.J.S., and J.D.J. performed data analysis. S.M.T.W., S.C., C.I., B.M., M.A.G., S.N.A., D.J.S., and J.D.J. worked on manuscript preparation.

ACKNOWLEDGMENTS

We thank D. Baker, M. Muse, P. Almeida Powell, and the staff of the Surrey Clinical Research Centre for their expert assistance in running the study. This study was funded by the UK Biotechnology and Biological Sciences Research Council (grants BB/I008470/1 and BB/J014451/1). B.M. and D.J.S. are co-directors of Stockgrand Ltd. and Surrey Assays Ltd.

Received: February 19, 2017
Revised: April 9, 2017
Accepted: April 27, 2017
Published: June 1, 2017

REFERENCES

1. Bass, J. (2012). Circadian topology of metabolism. Nature *491*, 348–356.
2. Johnston, J.D., Ordovás, J.M., Scheer, F.A., and Turek, F.W. (2016). Circadian rhythms, metabolism, and chrononutrition in rodents and humans. Adv. Nutr. *7*, 399–406.
3. Schibler, U., Gotic, I., Saini, C., Gos, P., Curie, T., Emmenegger, Y., Sinturel, F., Gosselin, P., Gerber, A., Fleury-Olela, F., et al. (2015). Clock-talk: interactions between central and peripheral circadian oscillators in mammals. Cold Spring Harb. Symp. Quant. Biol. *80*, 223–232.
4. Roenneberg, T., and Merrow, M. (2016). The circadian clock and human health. Curr. Biol. *26*, R432–R443.

5. Potter, G.D., Skene, D.J., Arendt, J., Cade, J.E., Grant, P.J., and Hardie, L.J. (2016). Circadian rhythm and sleep disruption: causes, metabolic consequences, and countermeasures. Endocr. Rev. *37*, 584–608.
6. Lucas, R.J., Peirson, S.N., Berson, D.M., Brown, T.M., Cooper, H.M., Czeisler, C.A., Figueiro, M.G., Gamlin, P.D., Lockley, S.W., O'Hagan, J.B., et al. (2014). Measuring and using light in the melanopsin age. Trends Neurosci. *37*, 1–9.
7. Lockley, S.W., Arendt, J., and Skene, D.J. (2007). Visual impairment and circadian rhythm disorders. Dialogues Clin. Neurosci. *9*, 301–314.
8. Damiola, F., Le Minh, N., Preitner, N., Kornmann, B., Fleury-Olela, F., and Schibler, U. (2000). Restricted feeding uncouples circadian oscillators in peripheral tissues from the central pacemaker in the suprachiasmatic nucleus. Genes Dev. *14*, 2950–2961.
9. Stokkan, K.A., Yamazaki, S., Tei, H., Sakaki, Y., and Menaker, M. (2001). Entrainment of the circadian clock in the liver by feeding. Science *291*, 490–493.
10. Mendoza, J., Graff, C., Dardente, H., Pevet, P., and Challet, E. (2005). Feeding cues alter clock gene oscillations and photic responses in the suprachiasmatic nuclei of mice exposed to a light/dark cycle. J. Neurosci. *25*, 1514–1522.
11. Johnston, J.D. (2014). Physiological responses to food intake throughout the day. Nutr. Res. Rev. *27*, 107–118.
12. Van Cauter, E., Polonsky, K.S., and Scheen, A.J. (1997). Roles of circadian rhythmicity and sleep in human glucose regulation. Endocr. Rev. *18*, 716–738.
13. Morgan, L., Hampton, S., Gibbs, M., and Arendt, J. (2003). Circadian aspects of postprandial metabolism. Chronobiol. Int. *20*, 795–808.
14. Scheer, F.A., Hilton, M.F., Mantzoros, C.S., and Shea, S.A. (2009). Adverse metabolic and cardiovascular consequences of circadian misalignment. Proc. Natl. Acad. Sci. USA *106*, 4453–4458.
15. Arble, D.M., Bass, J., Behn, C.D., Butler, M.P., Challet, E., Czeisler, C., Depner, C.M., Elmquist, J., Franken, P., Grandner, M.A., et al. (2015). Impact of sleep and circadian disruption on energy balance and diabetes: a summary of workshop discussions. Sleep *38*, 1849–1860.
16. Otway, D.T., Mäntele, S., Bretschneider, S., Wright, J., Trayhurn, P., Skene, D.J., Robertson, M.D., and Johnston, J.D. (2011). Rhythmic diurnal gene expression in human adipose tissue from individuals who are lean, overweight, and type 2 diabetic. Diabetes *60*, 1577–1581.
17. Mäntele, S., Otway, D.T., Middleton, B., Bretschneider, S., Wright, J., Robertson, M.D., Skene, D.J., and Johnston, J.D. (2012). Daily rhythms of plasma melatonin, but not plasma leptin or leptin mRNA, vary between lean, obese and type 2 diabetic men. PLoS ONE *7*, e37123.
18. Archer, S.N., Viola, A.U., Kyriakopoulou, V., von Schantz, M., and Dijk, D.J. (2008). Inter-individual differences in habitual sleep timing and entrained phase of endogenous circadian rhythms of BMAL1, PER2 and PER3 mRNA in human leukocytes. Sleep *31*, 608–617.
19. Duffy, J.F., and Dijk, D.J. (2002). Getting through to circadian oscillators: why use constant routines? J. Biol. Rhythms *17*, 4–13.
20. Scheer, F.A., Morris, C.J., and Shea, S.A. (2013). The internal circadian clock increases hunger and appetite in the evening independent of food intake and other behaviors. Obesity (Silver Spring) *21*, 421–423.
21. Sargent, C., Zhou, X., Matthews, R.W., Darwent, D., and Roach, G.D. (2016). Daily rhythms of hunger and satiety in healthy men during one week of sleep restriction and circadian misalignment. Int. J. Environ. Res. Public Health *13*, 170.
22. Shea, S.A., Hilton, M.F., Orlova, C., Ayers, R.T., and Mantzoros, C.S. (2005). Independent circadian and sleep/wake regulation of adipokines and glucose in humans. J. Clin. Endocrinol. Metab. *90*, 2537–2544.
23. Kräuchi, K., Cajochen, C., Werth, E., and Wirz-Justice, A. (2002). Alteration of internal circadian phase relationships after morning versus evening carbohydrate-rich meals in humans. J. Biol. Rhythms *17*, 364–376.
24. Mistlberger, R.E., and Skene, D.J. (2005). Nonphotic entrainment in humans? J. Biol. Rhythms *20*, 339–352.
25. Lamia, K.A., Storch, K.F., and Weitz, C.J. (2008). Physiological significance of a peripheral tissue circadian clock. Proc. Natl. Acad. Sci. USA *105*, 15172–15177.
26. Sadacca, L.A., Lamia, K.A., deLemos, A.S., Blum, B., and Weitz, C.J. (2011). An intrinsic circadian clock of the pancreas is required for normal insulin release and glucose homeostasis in mice. Diabetologia *54*, 120–124.
27. Marcheva, B., Ramsey, K.M., Buhr, E.D., Kobayashi, Y., Su, H., Ko, C.H., Ivanova, G., Omura, C., Mo, S., Vitaterna, M.H., et al. (2010). Disruption of the clock components CLOCK and BMAL1 leads to hypoinsulinaemia and diabetes. Nature *466*, 627–631.
28. Perelis, M., Marcheva, B., Ramsey, K.M., Schipma, M.J., Hutchison, A.L., Taguchi, A., Peek, C.B., Hong, H., Huang, W., Omura, C., et al. (2015). Pancreatic β cell enhancers regulate rhythmic transcription of genes controlling insulin secretion. Science *350*, aac4250.
29. Dyar, K.A., Ciciliot, S., Wright, L.E., Biensø, R.S., Tagliazucchi, G.M., Patel, V.R., Forcato, M., Paz, M.I., Gudiksen, A., Solagna, F., et al. (2013). Muscle insulin sensitivity and glucose metabolism are controlled by the intrinsic muscle clock. Mol. Metab. *3*, 29–41.
30. Rakshit, K., Hsu, T.W., and Matveyenko, A.V. (2016). Bmal1 is required for beta cell compensatory expansion, survival and metabolic adaptation to diet-induced obesity in mice. Diabetologia *59*, 734–743.
31. Boivin, D.B., James, F.O., Wu, A., Cho-Park, P.F., Xiong, H., and Sun, Z.S. (2003). Circadian clock genes oscillate in human peripheral blood mononuclear cells. Blood *102*, 4143–4145.
32. Loboda, A., Kraft, W.K., Fine, B., Joseph, J., Nebozhyn, M., Zhang, C., He, Y., Yang, X., Wright, C., Morris, M., et al. (2009). Diurnal variation of the human adipose transcriptome and the link to metabolic disease. BMC Med. Genomics *2*, 7.
33. Arendt, J., Bojkowski, C., Folkard, S., Franey, C., Marks, V., Minors, D., Waterhouse, J., Wever, R.A., Wildgruber, C., and Wright, J. (1985). Some effects of melatonin and the control of its secretion in humans. Ciba Found. Symp. *117*, 266–283.
34. Lewy, A.J., Ahmed, S., Jackson, J.M., and Sack, R.L. (1992). Melatonin shifts human circadian rhythms according to a phase-response curve. Chronobiol. Int. *9*, 380–392.
35. Burgess, H.J., Revell, V.L., and Eastman, C.I. (2008). A three pulse phase response curve to three milligrams of melatonin in humans. J. Physiol. *586*, 639–647.
36. Burke, T.M., Markwald, R.R., McHill, A.W., Chinoy, E.D., Snider, J.A., Bessman, S.C., Jung, C.M., O'Neill, J.S., and Wright, K.P., Jr. (2015). Effects of caffeine on the human circadian clock in vivo and in vitro. Sci. Transl. Med. *7*, 305ra146.
37. Khalsa, S.B., Jewett, M.E., Cajochen, C., and Czeisler, C.A. (2003). A phase response curve to single bright light pulses in human subjects. J. Physiol. *549*, 945–952.
38. Cuesta, M., Cermakian, N., and Boivin, D.B. (2015). Glucocorticoids entrain molecular clock components in human peripheral cells. FASEB J. *29*, 1360–1370.
39. Salgado-Delgado, R., Angeles-Castellanos, M., Saderi, N., Buijs, R.M., and Escobar, C. (2010). Food intake during the normal activity phase prevents obesity and circadian desynchrony in a rat model of night work. Endocrinology *151*, 1019–1029.
40. Horne, J.A., and Ostberg, O. (1976). A self-assessment questionnaire to determine morningness-eveningness in human circadian rhythms. Int. J. Chronobiol. *4*, 97–110.
41. Roenneberg, T., Wirz-Justice, A., and Merrow, M. (2003). Life between clocks: daily temporal patterns of human chronotypes. J. Biol. Rhythms *18*, 80–90.
42. Buysse, D.J., Reynolds, C.F., 3rd, Monk, T.H., Berman, S.R., and Kupfer, D.J. (1989). The Pittsburgh Sleep Quality Index: a new instrument for psychiatric practice and research. Psychiatry Res. *28*, 193–213.
43. Beck, A.T., and Beamesderfer, A. (1974). Assessment of depression: the depression inventory. Mod. Probl. Pharmacopsychiatry *7*, 151–169.

44. Beck, A.T., Rial, W.Y., and Rickels, K. (1974). Short form of depression inventory: cross-validation. Psychol. Rep. *34*, 1184–1186.
45. Johns, M.W. (1991). A new method for measuring daytime sleepiness: the Epworth sleepiness scale. Sleep *14*, 540–545.
46. Johns, M.W. (1992). Reliability and factor analysis of the Epworth Sleepiness Scale. Sleep *15*, 376–381.
47. Wehrens, S.M., Hampton, S.M., Finn, R.E., and Skene, D.J. (2010). Effect of total sleep deprivation on postprandial metabolic and insulin responses in shift workers and non-shift workers. J. Endocrinol. *206*, 205–215.
48. Akerstedt, T., and Gillberg, M. (1990). Subjective and objective sleepiness in the active individual. Int. J. Neurosci. *52*, 29–37.
49. Horne, J.A., and Baulk, S.D. (2004). Awareness of sleepiness when driving. Psychophysiology *41*, 161–165.
50. Flint, A., Raben, A., Blundell, J.E., and Astrup, A. (2000). Reproducibility, power and validity of visual analogue scales in assessment of appetite sensations in single test meal studies. Int. J. Obes. Relat. Metab. Disord. *24*, 38–48.
51. Fraser, S., Cowen, P., Franklin, M., Franey, C., and Arendt, J. (1983). Direct radioimmunoassay for melatonin in plasma. Clin. Chem. *29*, 396–397.
52. Riad-Fahmy, D., Read, G.F., Gaskell, S.J., Dyas, J., and Hindawi, R. (1979). A simple, direct radioimmunoassay for plasma cortisol, featuring a 125I radioligand and a solid-phase separation technique. Clin. Chem. *25*, 665–668.
53. Sletten, T.L., Revell, V.L., Middleton, B., Lederle, K.A., and Skene, D.J. (2009). Age-related changes in acute and phase-advancing responses to monochromatic light. J. Biol. Rhythms *24*, 73–84.

STAR★METHODS

KEY RESOURCES TABLE

REAGENT or RESOURCE	SOURCE	IDENTIFIER
Biological Samples		
Serial human blood samples	This paper	N/A
Serial human white adipose tissue biopsies	This paper	N/A
Critical Commercial Assays		
Paxgene RNA Tube	PreAnalytiX	Cat# 762165
Human Insulin-Specific RIA	Merck Millipore	Cat# HI-14K
RNeasy Mini Kit	QIAGEN	Cat# 74106
AffinityScript Multi Temperature cDNA Synthesis Kit	Agilent Technologies UK	Cat# 200436
LabChip RNA 6000 Nano kit	Agilent Technologies UK	Cat# 5067-1511
Oligonucleotides		
Primer/probe sequences for *PER3* and *BMAL1*	[18]	https://www.ncbi.nlm.nih.gov/pmc/articles/PMC2398752/
Primer/probe sequences for *PER2*	This paper	N/A

CONTACT FOR REAGENT AND RESOURCE SHARING

Further information and requests for resources and reagents should be directed to and will be fulfilled by the Lead Contact, Jonathan D. Johnston (j.johnston@surrey.ac.uk).

EXPERIMENTAL MODEL AND SUBJECT DETAILS

Ten male participants, 18-30 years old, were recruited to meet the following inclusion criteria: 20 $\geq$ BMI $\leq$ 30 kg/m^2 and fat mass $\geq$ 14%, Horne-Östberg (HÖ) questionnaire [40] 30 > HÖ < 70, completed Munich Chronotype Questionnaire (MCTQ) [41], Pittsburgh Sleep Quality Index (PSQI) [42] $\leq$ 5, Beck Depression Inventory (BDI) [43, 44] $\leq$ 9 and Epworth Sleepiness Scale (ESS) [45, 46] $\leq$ 9. The recruited participants had average age and BMI of 22.9 ± 1.27 and 23.1 ± 0.80 (mean ± SEM), respectively. Participants had regular sleep patterns on 5 or more nights per week (bed time 22:00-01:00 hr, wake up time 06:00-09:00 h), a sleep duration of 7-9 hr and habitual caffeine intake $\leq$ 300 mg/day. Absence of cotinine and drugs of abuse was checked by a urine test, and absence of alcohol by a breath test. All participants were free of medical conditions and/or medication known to affect study parameters. They also had normal blood hematology, biochemistry and serology. All study procedures received a favorable ethical opinion from The University of Surrey Ethics Committee. The study and data processing were carried out in accordance with the Helsinki Declaration of 1975, as revised in 2008, and the UK Data Protection Act (1998). All participants gave written informed consent after the nature and possible consequences of the study were explained.

METHOD DETAILS

Pre-laboratory study period

Participants were required to keep a self-selected regular 8 hr sleep period for 10 days prior to the start of the laboratory protocol. Self-selected sleep periods were based on habitual sleep patterns, as reported in PSQI and MCTQ data. Participants were permitted a nap during a 4 hr afternoon window, asked to obtain morning natural light exposure, and required to confirm behavior using voicemail, sleep diaries and light-sensitive actiwatches, as described previously [47]. For 1 week prior to the study, participants were asked to complete a food diary, consume $\leq$ 100 mg caffeine in the first 3 hr after waking, and $\leq$ 2 drinks of alcohol per day. Breakfast, lunch and dinner were consumed 0.5, 5.5 and 10.5 hr after waking. For 72 hr prior to the laboratory session, food was provided for the participants to eat at home and eaten within the same time windows as the preceding 4 days. Participants were also asked to refrain from heavy exercise, alcohol and caffeine over these final 72 hr.

Laboratory study design

All participants undertook a 13-day laboratory protocol (Figure 1). Throughout the laboratory protocol, each participant wore an ActiwatchL (Cambridge Neurotechnology Ltd) on their non-dominant wrist in order to provide an objective analysis of sleep markers during the sleep opportunities. Upon admission, continuing eligibility was assessed and repeat tests performed for breath alcohol, cotinine and drugs of abuse. During days 0-3 participants remained on their self-selected sleep-wake cycle and received meals 0.5,

5.5 and 10.5 hr after waking up. Breakfast, lunch and dinner were identical in energy and macronutrient content, with energy requirements determined using the Schofield equation. The macronutrient content of the meals was: 55% carbohydrate, of which 15% was sugars; 15% protein; and 30% fat, of which 11% was saturated. Participants slept in individual bedrooms. During the day participants were free to move around in bright room light (~500 lux in direction of gaze), but were not permitted to undergo any excessive exercise and were predominantly seated. Meals were eaten in the individual bedrooms to eliminate the impact of the smell and sight of other participants' food. On day 4, participants were cannulated after waking and remained in dim light until they began a 37 hr constant routine (CR1) in individual rooms. During the constant routine, participants were kept awake in a semi-recumbent posture in dim light (< 8 lux in the direction of gaze) and received hourly isocaloric sandwiches and milkshakes. Individual energy requirements during the constant routine were again determined using the Schofield equation. Participants were allowed to consume ≤ 100 mL water with each hourly meal except in the 10 min preceding a sample. A modified version of the Karolinska Sleepiness Scale [48, 49] followed by visual analog appetite scales [50] were completed before each meal. After the constant routine, participants had a standard night's sleep and underwent 6 more days of the sleep/wake light/dark cycle (day 6-11). Conditions were equal to days 1-3 except for a 5 hr delay in all meal times. A second 37 hr constant routine (CR2) then commenced on the morning of day 12, following the same procedure as for the earlier constant routine.

Biopsy and blood sample collection

To allow for a 'wash out' of any pre-constant routine effects of sleep, posture and food, sampling started at least 5.5 hr after the start of the constant routine. Using a modified version of our previous method [16], five serial gluteal subcutaneous white adipose tissue (WAT) biopsies were collected every 6 hr into cryotubes, frozen within 5 min in liquid nitrogen and stored in −80°C. Blood samples were taken via a cannula over a 32 hr period. Blood was collected hourly into lithium heparin vacutainers for analysis of melatonin and cortisol, as well as into di-potassium EDTA vacutainers for measurement of glucose, lipids and additional hormones. Immediately upon sample collection, vacutainers were inverted 10 times and cooled until centrifugation (1620 g at 4°C for 10 min, within 30 min of collection). The plasma fraction was transferred to microcentrifuge tubes, within 50 min of collection, and stored at −20°C. Blood samples for leukocyte clock gene expression were taken 2-hourly in PAXgene Blood RNA tubes (PreAnalytiX), as previously described [18].

Actigraphy measurements

Data from actiwatches were down-loaded and analyzed using CNT Sleep Analysis software (Cambridge Neurotechnology Ltd, Papworth Everard UK). Specific parameters assessed were: sleep duration, sleep efficiency, sleep latency and fragmentation index. For each participant, the average of each parameter was calculated for the 3 nights before each constant routine to represent that individual's sleep when experiencing early and late meal times. Data from one of the ten participants were excluded due to abnormal baseline values reported by the actiwatch.

Plasma hormone and metabolite measurements

Glucose and triglycerides were measured by enzymatic colorimetric detection in the Ilab (Instrumentation Laboratory, Warrington, UK) and hormones (melatonin, cortisol and insulin) were measured as described elsewhere [47, 51, 52]. Inter-assay CVs were < 10% for glucose and TAG; between 8.1 and 12.8% for melatonin; between 7.5 and 11.0% for cortisol; and < 20% for insulin.

Gene expression measurements

RNA was extracted from approximately 100 mg adipose tissue using the RNeasy mini kit (QIAGEN Ltd, Crawley, UK) according to the manufacturer's instructions. Leukocyte total RNA was extracted as previously described [18]. The RNA concentration and purity of each sample was assessed using a NanoDrop 2000 spectrophotometer (Thermo Scientific, Massachusetts, USA) and the integrity was measured with the Agilent 2100 Bioanalyzer LabChip RNA 6000 Nano kit (Agilent Technologies UK Ltd, Cheadle, UK) in approximately 15% of samples. RNA was then stored at −80°C. cDNA was made by random-primed reverse transcription of 100 ng adipose RNA or 200 ng leukocyte RNA with the AffinityScript Multi Temperature cDNA Synthesis Kit (Agilent) at a synthesis temperature of 42°C. cDNA was stored at −20°C.

We initially chose to analyze *PER3* and *BMAL1*, as these are representative of the 'positive' and 'negative' clock gene loops, and the genes that exhibited the most robust whole blood circadian rhythms in our hands [18]. We also analyzed the related canonical clock gene *PER2* in WAT samples. RT q-PCR was carried out with the Brilliant III Ultra-Fast master mix and ROX reference dye (Agilent, Mx3005P). *PER2* primers and probes were 5′-AAGCCCACATCACATCTCC-3′ (*PER2*-Forward), 5′-CACTGCACCCCTG AAAATAC-3′ (*PER2*-Reverse), [FAM]ACTCAGTCTGACAGCTTGCGACTGCAT[BHQ1] (*PER2*-Probe). All other TaqMan gene specific probes and primers have been reported previously [18]. All samples were run in triplicate. The thermal profile consisted of 3 min at 95°C followed by 15 s at 95°C and 20 s at 60°C (40 cycles). Ct values were calculated with an automated, amplification based threshold, adaptive baseline and moving average in MxPro v4.10 (Stratagene). Relative gene expression was then calculated according to the ΔΔCt method. Within each triplicate outliers were removed if the difference between replicate and triplicate mean exceeded 2SD of differences within a gene.

QUANTIFICATION AND STATISTICAL ANALYSIS

The phase of the melatonin rhythms was calculated as the 25% dim light melatonin onset (DLMO), i.e., the time at which melatonin reaches 25% of the peak concentration [53]. Melatonin and cortisol data were plotted relative to clock time, whereas all other data were plotted relative to each individual's DLMO in that constant routine. Data were grouped into time bins, the sizes relative to the sampling frequency for that parameter; 1 hr for melatonin, cortisol and subjective measures, 2 hr for blood gene expression, plasma glucose, TAG and insulin, and 4 hr for adipose gene expression.

Analysis of all temporal profiles was first carried out using a 2-way repeated-measures ANOVA, with time of day and meal schedule as the two independent variables, both of which were repeated-measures. Circadian phase for melatonin in each participant was assessed using DLMO, as described above. Phase assessment for other parameters was estimated by deriving acrophases (peak times) from cosinor analysis. The effect of the delayed meals on that measurement's acrophase was assessed by a paired t test of individual phase; the test was one-tailed when a one-directional effect, i.e., a delay, had been hypothesized.

Paired t tests were also used to compare the average concentrations of plasma glucose, insulin and triglyceride. The peak concentration of a glucose rhythm was estimated by calculating the mean average of the numerically highest value within each data series and its two immediately adjacent time points. Similarly, the lowest concentration was estimated by averaging the numerically lowest value and its two immediately adjacent time points. Average peak and trough concentrations in the two constant routines were analyzed by 2-way repeated-measures ANOVA.

In order to minimize type 1 statistical errors (i.e., false positives), Bonferroni corrections were applied to critical p values. For analysis of plasma molecules (melatonin, cortisol, glucose, insulin, triglyceride), a correction factor of 5 was employed, resulting in a critical p value of 0.01. For analysis of adipose gene expression (*PER2*, *PER3*, *BMAL1*), a correction factor of 3 was employed, resulting in a critical p value of 0.017.

Data are provided as both grouped and individual values. Grouped data are presented as mean ± SEM, with relevant n values described in the figure legends. Analyses were performed using Graphpad Prism 7.0 software.

Current Biology, Volume *27*

Supplemental Information

Meal Timing Regulates the Human Circadian System

Sophie M.T. Wehrens, Skevoulla Christou, Cheryl Isherwood, Benita Middleton, Michelle A. Gibbs, Simon N. Archer, Debra J. Skene, and Jonathan D. Johnston

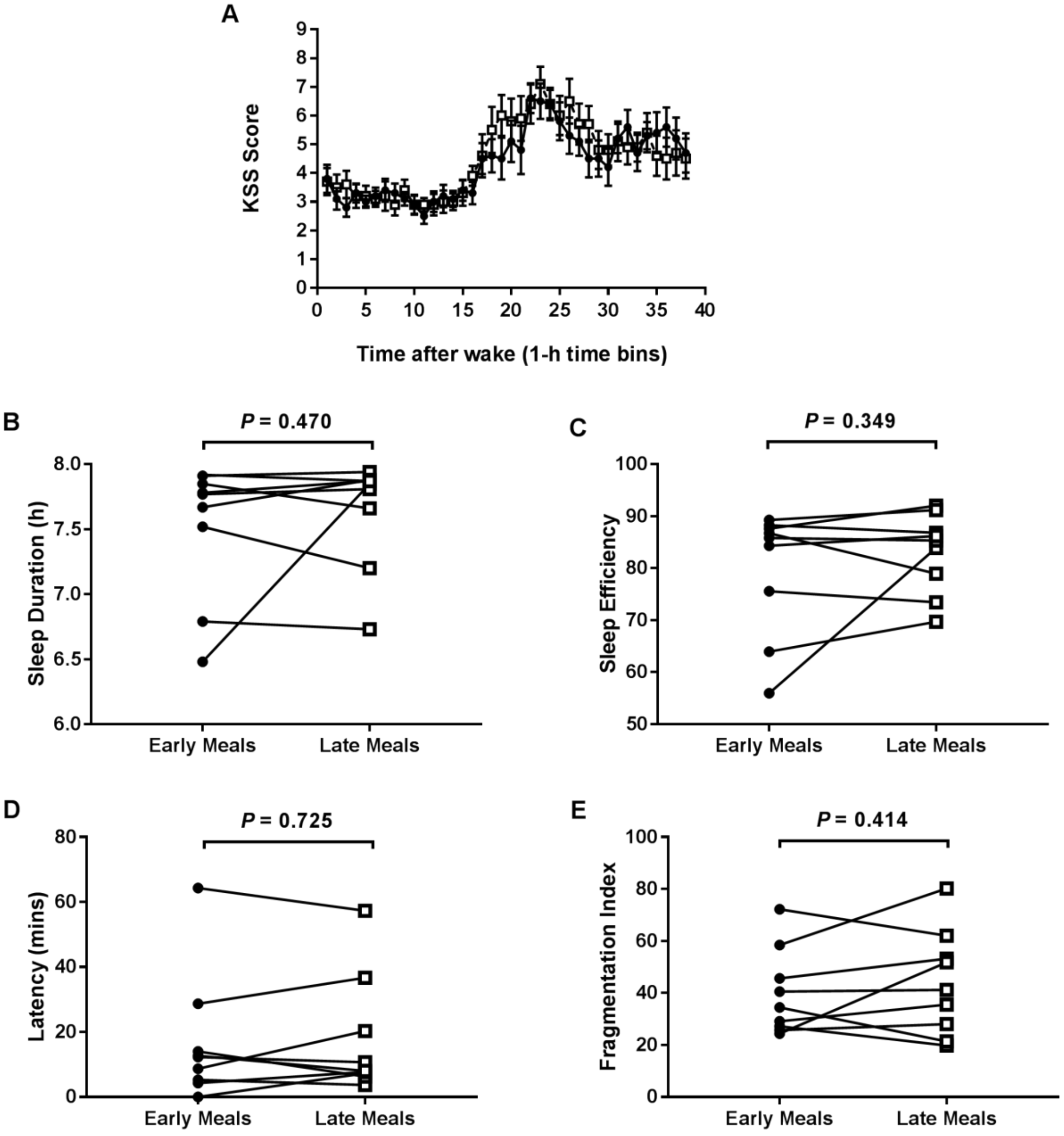

Figure S1. Sleep markers are not altered by a 5-hour delay in meal times (related to Figure 1).
(A) Subjective sleepiness was determined by hourly completion of the Karolinska Sleepiness Scale (KSS) during constant routine conditions. Black circles with solid lines represent data following early meals (0.5, 5.5 and 10.5 hours after waking up). White squares with dashed lines represent data following a 5-hour delay in each meal. There was a significant effect of time ($F_{(37,333)}$ = 10.84, p < 0.001) , but no effect of meals ($F_{(1,9)}$ = 0.36, p = 0.562) or meal x time interaction ($F_{(37,333)}$ = 1.14, p = 0.274; 2-way repeated measures ANOVA). Data are mean ± SEM, n = 10, plotted as KSS score relative to each individual's time since wake.
(B-E) Actigraphy analysis of sleep periods following early and late meals. Data are from n = 9 participants and analysed by paired t-test. There was no significant effect on (B) sleep duration ($t_{(8)}$ = 0.759, p = 0.470). (C) sleep efficiency ($t_{(8)}$ = 0.995, p = 0.349). (D) sleep latency ($t_{(8)}$ = 0.365, p = 0.725). or (E) fragmentation index ($t_{(8)}$ = 0.862, p = 0.414).

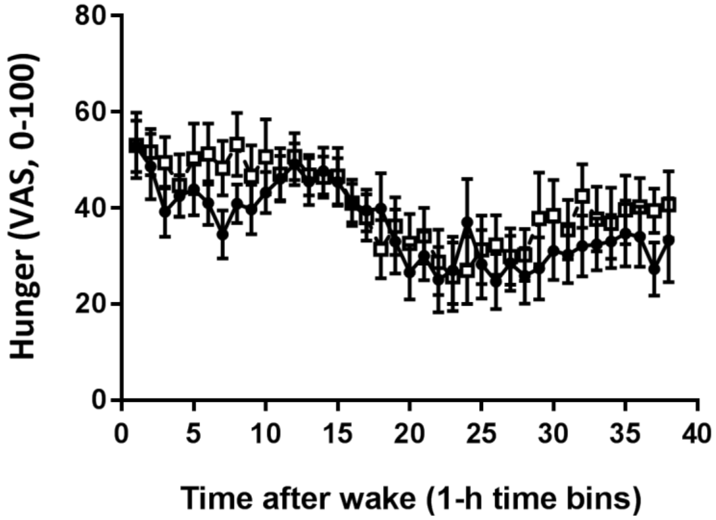

Figure S2. Subjective hunger is not altered by a 5-hour delay in meal times (related to Figure 1).
Hunger was determined by hourly completion of a hunger visual analogue scale (VAS) during constant routine conditions. Black circles with solid lines represent data following early meals (0.5, 5.5 and 10.5 hours after waking up). White squares with dashed lines represent data following a 5-hour delay in each meal. There was a significant effect of time ($F_{(37,333)} = 6.12$, $p < 0.001$), but no significant effect of meal ($F_{(1,9)} = 1.60$, $p = 0.238$) or meal x time interaction ($F_{(37,333)} = 1.03$, $p = 0.424$; 2-way repeated measures ANOVA). Data are mean ± SEM, n = 10 , plotted as hunger score relative to each individual's time since wake.

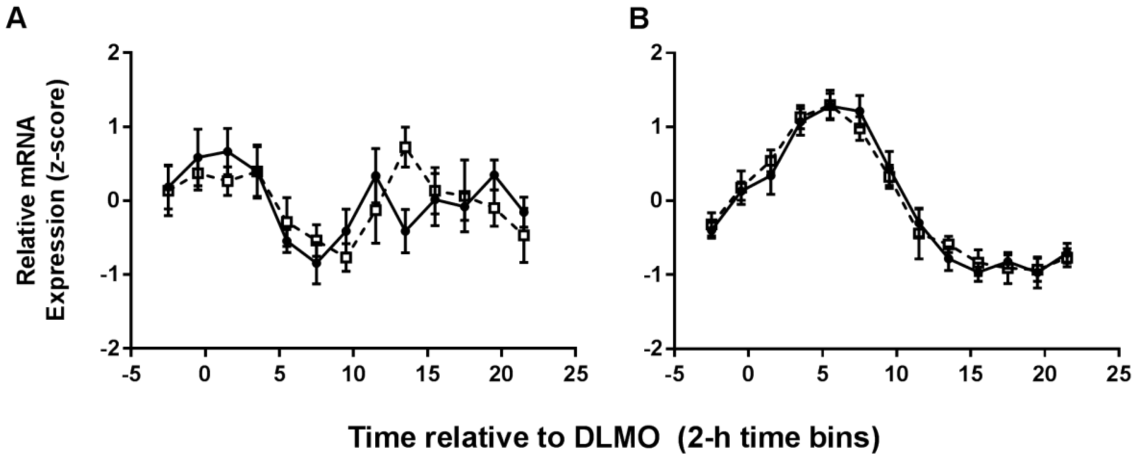

Figure S3. Clock gene rhythms in whole blood are not altered by a 5-hour delay in meal times (related to Figure 3).
Temporal expression profiles of (A) *BMAL1* and (B) *PER3* in 2-hourly blood leukocyte samples collected in constant routine conditions. Black circles with solid lines represent data following early meals (0.5, 5.5 and 10.5 hours after waking up). White squares with dashed lines represent data following a 5-hour delay in each meal. Data are mean ± SEM, n = 10, plotted as z-scored relative mRNA expression relative to each individual's dim light melatonin onset (DLMO).
